# Associations Between Sleep Health and Diet Quality and Dietary Intake During Pregnancy: Evidence From Two Australian Cohorts

**DOI:** 10.1111/mcn.70256

**Published:** 2026-09-21

**Authors:** Genette Li Hui Chua, Mitch J. Duncan, Clare E. Collins, Rachael M. Taylor, Kee June Ooi, Craig E. Pennell, Tegan Grace, Poonam Kaur Pannu, Sasha Fenton

**Affiliations:** ^1^ School of Health Sciences, College of Health, Medicine and Wellbeing University of Newcastle, University Drive, Callaghan New South Wales Australia; ^2^ School of Medicine and Public Health; College of Health, Medicine and Wellbeing, The University of Newcastle University Drive, Callaghan New South Wales Australia; ^3^ Centre for Active Living and Learning University of Newcastle, Callaghan New South Wales Australia; ^4^ Food and Nutrition Research Program, Hunter Medical Research Institute New Lambton Heights New South Wales Australia; ^5^ Mothers and Babies Research Program, Hunter Medical Research Institute New Lambton Heights New South Wales Australia; ^6^ Maternity and Gynaecology Department John Hunter Hospital Newcastle New South Wales Australia; ^7^ The Kids Research Institute Australia, Nedlands Western Australia Australia; ^8^ Curtin University Bentley Western Australia Australia; ^9^ The University of Western Australia, Nedlands Western Australia Australia

**Keywords:** diet quality, dietary intake, NEW1000, ORIGINS, pregnancy, sleep duration, sleep quality

## Abstract

Poor sleep and suboptimal diet are common during pregnancy and contribute to adverse maternal and infant outcomes. While sleep‐diet associations are documented in general populations, evidence in pregnancy is scarce. This study investigated associations between sleep quality and duration and diet quality and dietary intake among Australian pregnant women. Data from two Australian pregnancy cohorts (Newcastle1000 and ORIGINS; *n* = 1070) were collected at 18–20 weeks gestation (2017–2024). Sleep was assessed using the Pittsburgh Sleep Quality Index (PSQI), and dietary intake and diet quality using the Australian Eating Survey and Australian Recommended Food Score (ARFS). Generalised linear models examined associations between sleep quality (continuous PSQI score) and sleep duration (< 7 h, 7–9 h, > 9 h) and dietary variables, adjusting for age, income, pre‐pregnancy BMI, mental health, physical activity, and cohort. Participants were aged 32.3 ± 4.3 years; with a pre‐pregnancy BMI of 25.9 ± 5.7 kg/m^2^ and 95.3% were English‐speaking. Most participants reported poor sleep quality (72.9%; PSQI global score 6.4 ± 2.8) and suboptimal diet quality (ARFS 35.5 ± 8.9). Poor sleep quality was associated with higher saturated fat intake (Exp(β) = 1.004, 95% CI: 1.000,1.008, *p* = 0.046). Short sleep duration (< 7 h) was associated with lower diet quality (*β* = −2.60, 95% CI: −3.95, −1.25, *p* < 0.001) and lower fibre intake (Exp(β) = 0.945, 95% CI: 0.900,0.992, *p* = 0.022). No other significant associations were observed in adjusted models. Poor sleep quality and short sleep duration were associated with less favourable dietary patterns during pregnancy. This highlights the need for further research to inform integrated interventions that consider sleep quality and duration alongside strategies to support healthier eating patterns and improve maternal and infant health outcomes.

## Summary

- Poor sleep and suboptimal diet are prevalent during pregnancy and contribute to adverse maternal and infant health outcomes; however, associations between sleep and dietary behaviours during pregnancy are relatively underexplored.
- This study found that poor sleep quality was associated with higher total saturated fat intake, while short sleep (< 7 h/night) was linked with lower diet quality and lower total fibre intake.
- These findings contribute to the limited evidence on associations between sleep quality, sleep duration, and dietary outcomes during pregnancy, and highlight the importance of considering sleep alongside nutrition and lifestyle factors in antenatal care. Further research is needed to clarify the direction and causality of these relationships.

## 1 Introduction

Sleep duration and sleep quality are increasingly recognised as behavioural determinants of dietary behaviours; however, their role during pregnancy remains underexplored (Soltanieh et al. 2021; von Ash et al. 2023). Sleep health encompasses multiple dimensions including duration, efficiency, timing, alertness, and overall quality, with each linked to health outcomes such as obesity, diabetes and cardiovascular disease (Buysse 2014). Pregnancy introduces unique challenges to sleep, with hormonal fluctuations, physical discomfort, and nocturnal symptoms such as gastroesophageal reflux and nocturia contributing to widespread sleep disturbances (Kember et al. 2023; Silvestri and Aricò 2019)). Prevalence estimates suggest that approximately 60% of pregnant women experience poor sleep quality, and up to 40% report short sleep duration (Mislu et al. 2024; Senaratna et al. 2023). These sleep disturbances may influence dietary behaviours through mechanisms such as increased hedonic drive for palatable foods, extended eating windows, and impaired appetite regulation, as observed in non‐pregnant populations (Akhlaghi and Kohanmoo 2025; Fenton et al. 2021).

Diet quality refers to how closely an individual's overall food intake aligns with dietary guidelines, reflecting adequacy and variety (Fenton et al. 2024). During pregnancy, diet quality is critical for maternal and foetal health (Abdollahi et al. 2021; Marshall et al. 2022). Dietary patterns describe the overall combination, variety and frequency of foods habitually consumed, providing a broader representation of dietary intake beyond individual nutrients or foods (Schulze et al. 2018). Healthier dietary patterns are associated with higher diet quality and reduced risks of adverse health consequences such as gestational diabetes, hypertension, and preterm birth (Kibret et al. 2018; Kinshella et al. 2021). Globally, however, few pregnant women achieve national dietary recommendations (Caut et al. 2020; Fenton et al. 2025; Pannu et al. 2024). A systematic review of 18 studies across 10 countries reported poor alignment with dietary guidelines in women during preconception and pregnancy phases (Caut et al. 2020). Understanding behavioural factors that influence food selection during pregnancy is essential for designing interventions that improve maternal nutrition and reduce adverse health outcomes.

Growing evidence in general populations suggests that sleep influences dietary intake (Al Khatib et al. 2017; Fenton et al. 2021). A meta‐analysis of intervention studies found that short sleep duration (≤ 5.5 h) increases daily energy intake, including carbohydrate, protein and fat intake (Fenton et al. 2021). Evidence on sleep quality and diet is more limited, but some studies indicate that poorer sleep quality is associated with higher consumption of added sugars (Zuraikat et al. 2020), confectionary and sugar‐sweetened beverages (Katagiri et al. 2014). Research in pregnant women, however, remains limited and methodologically heterogeneous (von Ash et al. 2023). A recent systematic review identified only 25 studies examining sleep‐diet associations during pregnancy, which varied widely in measures, timing, and outcomes, with few employing validated tools for assessing both sleep and diet (von Ash et al. 2023). This lack of consistency limits the ability to draw strong conclusions, leaving a critical gap in understanding how sleep may influence dietary intake and diet quality during pregnancy.

This study aimed to assess the association between sleep quality and duration and diet quality and dietary intake during pregnancy in a contemporary sample of Australian women. By examining behavioural links between sleep and dietary intake, this research may address an important evidence gap regarding sleep quality, sleep duration, and dietary intake during pregnancy.

## 2 Methods

### 2.1 Study Design and Ethics

The current study is a secondary analysis involving cross‐sectional data from two cohorts of pregnant women. Participants were recruited via two hospital antenatal clinics in New South Wales (NSW) and Western Australia (WA), Australia.

Ethics approval was obtained from the Hunter New England Human Research Ethics Committee (2020/ETH02881) for the Newcastle1000 (NEW1000) cohort, and the Human Research Ethics Committee of Joondalup Health Campus (2023/ETH/0001) for the ORIGINS cohort.

### 2.2 Participants

Pregnant women who attended the routine antenatal clinics at John Hunter Hospital and Joondalup Health Campus were recruited. The criteria for inclusion in the analyses were: (1) pregnant women aged ≥ 18 years; (2) carrying a singleton pregnancy; (3) had complete dietary and sleep data. Participants were excluded if they did not meet these criteria, specifically if they were aged < 18 years, had a multiple pregnancy, or had missing dietary or sleep data. Written informed consent was obtained from all participants.

### 2.3 Study Recruitment

#### 2.3.1 NEW1000

NEW1000 is a longitudinal cohort study based in Newcastle, NSW, Australia, involving recruitment and assessment of 1000 families over 3 years, with in‐person assessments, biological sampling, foetal ultrasound scans and online surveys (Grace et al. 2023). NEW1000 is a collaboration between John Hunter Hospital, the Hunter Medical Research Institute and the University of Newcastle. Eligible participants included pregnant women aged ≥ 18 years (or younger with parental consent) attending the John Hunter Hospital antenatal clinic, who intended to reside in the study region for long‐term follow‐up. Exclusion criteria included: high dependence on medical care (including recent intensive care unit (ICU) admission or life‐ limiting comorbidity), surrogate pregnancies where the biological mother is not the child's legal guardian, cognitive impairment preventing completion of data collection, provision of informed consent or understanding of written/verbal instructions, and lack of parental consent for the child's participation. Full details are described elsewhere (Grace et al. 2023). Participants were recruited between 2021 and 2024 at approximately 14 weeks gestation and provided data for the current study at approximately 20 weeks gestation.

#### 2.3.2 ORIGINS

ORIGINS is a longitudinal cohort study and has recruited over 10,000 birthing families from Perth, WA, Australia (D'Vaz et al. 2023; Silva et al. 2020). Participants were eligible if they were pregnant women planning to deliver at Joondalup Health Campus, including both public and private patients. Full details are described elsewhere (Silva et al. 2020). From the total sample, 4000 participants consented to provide biological samples, complete questionnaires and attend clinic appointments. ORIGINS is a collaboration between the Joondalup Health Campus and The Kids Research Institute Australia. Participants included in the current analyses were recruited and provided data at the Joondalup Health Campus between 2017 and 2022 at 18–20 weeks' gestation (D'Vaz et al. 2023; Silva et al. 2020). The families are followed for sample collections, paediatric appointments and questionnaires until the child is in early childhood. Detailed recruitment processes for NEW1000 and ORIGINS are described elsewhere (D'Vaz et al. 2023; Grace et al. 2023; Silva et al. 2020).

### 2.4 Data Collection

#### 2.4.1 Sleep Quality and Sleep Duration

Sleep quality and sleep duration were measured using the Pittsburgh Sleep Quality Index (PSQI), a validated tool used to assess sleep over the past month (Buysse et al. 1989; Mollayeva et al. 2016). The PSQI consists of 19 items derived from seven components that assess different aspects of sleep, including subjective sleep quality, sleep duration, sleep latency, sleep disturbances, habitual sleep efficiency, use of sleeping medications and daytime dysfunction (Buysse et al. 1989). Each component is scored on a 0–3 scale, and the sum of seven component scores produces a global score ranging from 0 to 21, with higher scores indicating poorer sleep quality (Buysse et al. 1989). Participants with a score < 5 are classified as good sleepers, while those with a score ≥ 5 are considered poor sleepers (Buysse et al. 1989). The PSQI global score was used to capture overall sleep quality across multiple correlated domains, rather than analysing individual subcomponents separately. Sleep duration was derived from the PSQI item asking participants to report their average hours of actual sleep per night over the past month (Buysse et al. 1989).

#### 2.4.2 Dietary Intake

Dietary intake was assessed using the Australian Eating Survey (AES), a 120‐item semi‐quantitative food frequency questionnaire (FFQ) (C.E. Collins et al. 2015). The AES assesses consumption frequency of food and beverages over the previous three to 6 months on a Likert scale with response options ranging from ‘never’ up to ‘7 times per day’. Questions relate to fruit, vegetables, grain foods, dairy foods, main meals, snack foods, beverages, and other foods (C. E. Collins et al. 2014). The AES also contains 15 supplementary questions including age, use of nutritional supplements and dietary intake‐related behaviours, e.g. takeaway food consumption (C. E. Collins et al. 2014). Standard portion sizes for each food item were derived from the most recent National Nutrition Survey data (Australian Bureau of Statistics 1995). Nutrient intakes from the AES were computed using data in the Australian Food and Nutrient (AUSNUT) 2011–13 database (Food Standards Australia and New Zealand (FSANZ) 2023). Total energy intake (kJ/day) was calculated by multiplying the reported consumption frequency of each food item by the portion size, with nutrient composition data derived from the AUSNUT 2011–13 database. Kilojoules from all food and beverage items were summed to generate total daily energy intake. The proportion of total energy intake derived from nutrient‐dense core foods (e.g. fruit, vegetables) and energy‐dense, non‐core foods high in saturated fat, added sugars, added salt (e.g. takeaway foods) and/or alcohol were calculated. The AES has been validated in Australian adults and demonstrated a high level of accuracy in estimating nutrient, fruit and vegetable intakes (Burrows et al. 2015; Schumacher et al. 2016). It has also been implemented across five Australian pregnancy cohort studies including NEW1000 (Grace et al. 2023), ORIGINS (Silva et al. 2020), Baby1000 (Grech et al. 2023), Queensland Family Cohort (Wilkinson et al. 2022), and Gomeroi Gaaynggal (Lee et al. 2019).

#### 2.4.3 Diet Quality

Diet quality was assessed using the Australian Recommended Food Score (ARFS), a food‐based diet quality score calculated to assess diet quality as a single continuous variable (C. E. Collins et al. 2015; Collins et al. 2008). The ARFS is derived from a subset of 70 AES questions, measuring dietary variety within nutrient‐dense core food groups recommended in the Australian Guide to Healthy Eating (AGHE) (Australian Government National Health and Medical Research Council 2017). The ARFS includes eight sub‐scales, with 20 questions related to vegetables, 12 to fruit, 13 to protein foods (seven to meat and six to vegetarian protein sources), 12 to breads and cereals, 10 to dairy foods, one to water and two to spreads/sauces (C. E. Collins et al. 2015; Collins et al. 2008). Most food items are awarded one point if consumed at least once per week, with adjustment for red meat and some dairy foods (e.g. frozen yoghurt, ice‐cream), where a limit is applied for higher intakes associated with higher saturated fat intake and disease risk. Extra points are awarded for higher consumption of vegetables and healthier types of bread and milk (C. E. Collins et al. 2015). Participants who indicate they follow a vegetarian eating pattern are assigned a zero score for the meat subscale, with double points awarded for each of the meat alternatives questions. An additional point is also awarded if soybeans, tofu, other beans and lentils were reported as consumed once per week or more. Points are summed for each item to give a total ARFS score ranging from zero to 73, with higher scores reflecting higher diet quality (C. E. Collins et al. 2015). The ARFS is categorised as: ‘needs work’ (Score: < 33), ‘getting there’ (Score: 33–38), ‘excellent’ (Score: 39–46), or ‘outstanding’ (Score: 47+) (Williams et al. 2017). ARFS validation studies reported high reliability and consistency in evaluating diet quality and estimating fruit and vegetable intakes among Australian adults (Ashton et al. 2017; C.E. Collins et al. 2014).

#### 2.4.4 Sociodemographic and Health Characteristics

Sociodemographic and health data including age, birth country, Aboriginal or Torres Strait Islander status, language/s spoken, household income, relationship status, postcode, height, and pre‐pregnancy body weight were self‐reported via online surveys. Self‐reported height and body weight were used to calculate pre‐pregnancy BMI. Smoking status was sourced from hospital medical records. Mental health indicators, including depression, anxiety and stress, were self‐reported via the validated Depression Anxiety Stress Scales‐21 items (DASS‐21), with normal scores ranging from 0 to 9 points, 0–7 points and 0–14 points, respectively (Henry and Crawford 2005). Physical activity was self‐reported via the validated International Physical Activity Questionnaire (IPAQ) short‐form and reported as MET‐mins per week and physical activity level (low, moderate, high) (Craig et al. 2003). Residential postcodes were mapped to the Australian Bureau of Statistics' Socioeconomic Indexes for Areas (SEIFA), specifically the Index of Relative Socioeconomic Advantage and Disadvantage (IRSAD) quintiles which reflect the economic and social conditions of households within a geographical area (Australian Bureau of Statistics 2023). SEIFA scores served as an indicator for socioeconomic status (SES), with a lower score representing greater relative disadvantage, and a higher score indicating greater relative advantage (Australian Bureau of Statistics 2023).

### 2.5 Statistical Analysis

Participants reporting implausible energy intakes (< 4500 kJ/day or > 20,000 kJ/day) were excluded from the analyses to reduce bias from misreporting (Meltzer et al. 2008). Descriptive statistics (means, standard deviations, frequencies) were used to summarise participant characteristics, sleep duration and quality, and dietary intake and quality. Continuous variables were reported as means and standard deviations, and categorical variables as frequencies and percentages. To examine associations between sleep quality and sleep duration with dietary intake and quality during pregnancy, generalised linear models (GLM) were conducted with the PSQI global score and sleep duration per night as the independent variables. Separate models were run for each sleep exposure (quality and duration), with dietary intake variables treated as dependent variables. Sleep quality was examined as a continuous variable (Score: 0–21). Given the established U‐shaped curve between sleep duration and health outcomes, sleep duration was examined as a categorical variable: short sleep duration (< 7 h), normal sleep duration (7–9 h), long sleep duration (> 9 h), consistent with age‐specific sleep duration guidelines (Hirshkowitz et al. 2015). Model selection was informed by variable distributions and residual diagnostics.

The dietary variables assessed included: ARFS, total energy intake, percentage of energy intake from core foods, percentage of energy intake from non‐core foods, and percentage of energy intake from protein, carbohydrate, total fat, and saturated fats, fibre (grams), iron (mg), iodine (µg), zinc (mg), and dietary folate equivalents (µg). To examine associations between sleep and diet, both unadjusted and adjusted analyses were conducted, with coefficients, standard errors, and 95% confidence intervals (CI) reported. Adjusted models included the potential confounders of age, household income, pre‐pregnancy BMI, physical activity (MET‐min per week), DASS depression, anxiety and stress scores, and cohort, which were informed by prior literature (Fenton et al. 2025; McCarthy et al. 2023; Silva‐Perez et al. 2019). Model fit was evaluated through residual diagnostics, with no severe violations observed. Statistical significance was set at *p* < 0.05, with results reported as adjusted beta‐coefficients and 95% confidence intervals. Analyses were conducted using Jamovi statistical software 2.6.26 (The jamovi Project 2025).

### 2.6 Ethics Statement

This study was conducted according to the guidelines laid down in the Declaration of Helsinki. Procedures involving research study participants were approved by Ramsay Health Care WA | SA Human Research Ethics Committee (2023/ETH/0001) for the ORIGINS cohort, and the University of Newcastle Human Research Ethics Committee (H‐2023‐0239). Stage One of the NEW1000 Study was approved by the Hunter New England Human Research Ethics Committee (2020/ETH02881) on 8th December 2020, with an amendment for extension to Stage Two approved on 28thth July 2022. Governance of Stage Two was thereafter moved to the University of Newcastle Human Research Ethics Committee (H‐2025‐0230) as of 16th October 2025. All projects using NEW1000 Study data or biological samples are required to submit an initial application to the NEW1000 Executive Committee and have relevant ethics and safety clearances prior to initiation.

## 3 Results

Following the exclusion of 32 participants classified as misreporters of energy intake, 1070 pregnant women were included in the analyses.

### 3.1 Participant Sociodemographic, Health and Lifestyle Characteristics

The participants' sociodemographic, health and lifestyle characteristics are presented in Table 1.

**Table 1:** Participant sociodemographic, health and lifestyle characteristics.

Characteristic	Cohort		
	NEW1000 n = 615	ORIGINS n = 455	Total sample n = 1070
Age (years), Mean (SD)	32.1 (4.1)	32.6 (4.4)	32.3 (4.3)
Birth country, *n* (%)			
Australia	547 (88.9)	219 (48.1)	766 (71.6)
United Kingdom	14 (2.3)	118 (25.9)	132 (12.3)
New Zealand	9 (1.5)	17 (3.7)	26 (2.4)
Other/Not reported	45 (7.3)	101 (22.2)	146 (13.6)
Aboriginal or Torres Strait Islander, *n* (%)			
No	589 (95.8)	451 (99.1)	1,040 (97.2)
Yes	25 (4.1)	4 (0.9)	29 (2.7)
Not reported	1 (0.2)	0	1 (0.1)
Language spoken at home, *n* (%)			
English	599 (97.4)	421 (92.5)	1020 (95.3)
Other/Not reported	16 (2.6)	34 (7.5)	50 (4.7)
Annual household income (AUD$), *n* (%)			
≤ $50,000	16 (2.6)	12 (2.6)	28 (2.6)
$50,001–$100,000	98 (15.9)	97 (21.3)	195 (18.2)
$100,001–$150,000	155 (25.2)	181 (39.8)	336 (31.4)
≥ $150,001	237 (38.5)	165 (36.3)	402 (37.6)
Not reported	109 (17.7)	0	109 (10.2)
Socioeconomic status, *n* (%)			
IRSAD deciles 1–5	157 (25.5)	41 (9.0)	198 (18.5)
IRSAD deciles 6–10	456 (74.1)	414 (91.0)	870 (81.3)
Not reported	2 (0.3)	0	2 (0.2)
Pre‐pregnancy BMI (kg/m^2^), Mean (SD)	26.2 (6.1)	25.7 (5.2)	25.9 (5.7)
Pre‐pregnancy BMI classification (kg/m^2^), *n* (%)			
Underweight (< 18.5)	7 (1.1)	9 (2.0)	16 (1.5)
Healthy weight (≥ 18.5 to ≤ 24.9)	212 (34.5)	233 (51.2)	445 (41.6)
Overweight (≥ 25 to ≤ 29.9)	115 (18.7)	116 (25.5)	231 (21.6)
Obese (≥ 30)	88 (14.3)	89 (19.6)	177 (16.5)
Not reported	193 (31.4)	8 (1.8)	201 (18.8)
Smoked during pregnancy, *n* (%)			
No	606 (98.5)	452 (99.3)	886 (98.9)
Yes	9 (1.5)	3 (0.7)	12 (1.1)
Mental health indicators, *n* (%)			
Depression			
Normal	608 (98.9)	410 (90.1)	1,018 (95.1)
Mild/Moderate	7 (1.1)	41 (9.0)	48 (4.5)
Severe/Extremely severe	0	4 (0.9)	4 (0.4)
Anxiety			
Normal	595 (96.7)	385 (84.6)	980 (91.6)
Mild/Moderate	20 (3.3)	55 (12.1)	75 (7.0)
Severe/Extremely severe	0	15 (3.3)	15 (1.4)
Stress			
Normal	609 (99.0)	413 (90.8)	1022 (95.5)
Mild/Moderate	6 (1.0)	37 (8.1)	43 (4.0)
Severe/Extremely severe	0	5 (1.1)	5 (0.5)
Physical activity			
MET‐mins per week, mean (SD)	2097.0 (2668.7)	2105.3 (2226.9)	2100.5 (2490.5)
Physical activity level, *n* (%)			
Low	166 (27.0)	76 (16.7)	242 (22.6)
Moderate	310 (50.4)	358 (78.7)	668 (62.4)
High	139 (22.6)	21 (4.6)	160 (15.0)
Sleep quality			
Sleep quality score (0–21), Mean (SD)	6.3 (3.1)	6.4 (2.3)	6.4 (2.8)
Sleep quality category, *n* (%)			
Good quality (< 5)	199 (32.4)	91 (20.0)	290 (27.1)
Poor quality (≥ 5)	416 (67.6)	364 (80.0)	780 (72.9)
Sleep duration			
Hours/day, Mean (SD)	7.5 (1.1)	7.2 (1.2)	7.4 (1.2)
< 7 h/day, *n* (%)	110 (17.9)	116 (25.5)	226 (21.1)
7–9 h/day, *n* (%)	484 (78.7)	327 (71.9)	811 (75.8)
> 9 h/day, *n* (%)	21 (3.4)	12 (2.6)	33 (3.1)
Diet quality			
Diet quality score (0–73), mean (SD)	35.5 (8.9)	35.1 (9.2)	35.3 (9.0)
Dietary intake, mean (SD)			
Energy intake (kJ/day)	8466 (2375)	8801 (2381)	8609 (2382)
Core food intake (%EI)	65.5 (10.6)	66.5 (11.1)	65.9 (10.8)
Non‐core food intake (%EI)	34.5 (10.6)	33.5 (11.1)	34.1 (10.8)
Carbohydrates (%EI)	45.5 (5.7)	44.6 (5.9)	45.1 (5.8)
Protein (%EI)	17.3 (2.7)	17.8 (2.6)	17.5 (2.7)
Total fat (%EI)	36.9 (4.3)	37.4 (4.5)	37.1 (4.4)
Saturated fat (%EI)	13.7 (2.3)	14.4 (2.3)	14.0 (2.3)
Fibre (g/day)	28.3 (9.5)	27.6 (8.6)	28.0 (9.1)
Iron (mg/day)	10.5 (3.2)	10.7 (3.2)	10.6 (3.2)
Zinc (mg/day)	10.8 (3.4)	11.5 (3.5)	11.1 (3.4)
Iodine (µg/day)	119.6 (47.7)	136.7 (54.0)	126.9 (51.1)
Folate eq. (µg/day)	547.4 (188.6)	557.2 (181.3)	551.6 (185.5)

The women had a mean age of 32.3 (SD 4.3) years. Most participants (95.3%) spoke English at home, and the highest proportion of participants (37.6%) reported an annual household income ≥ $150,000 AUD. Most participants (81.3%) resided in areas classified within IRSAD deciles 6–10, indicating relatively higher socioeconomic advantage. The mean pre‐pregnancy BMI was 25.9 (SD 5.7) kg/m^2^, with almost half the participants (41.6%) within the healthy pre‐pregnancy BMI range category (i.e., 18.5–24.9 kg/m^2^). Most participants scored within the normal range for depression (95.1%), anxiety (91.6%) and stress (95.5%) symptoms, and 62.4% self‐reported being moderately physically active.

The average global PSQI score was 6.4 (SD 2.8) points out of 21, and 72.9% of participants reported poor sleep quality. The mean sleep duration was within the recommended range at 7.4 h (SD 1.2) per day, with 75.8% achieving 7–9 h sleep per day. Participants reported a mean ARFS (diet quality score) of 35.5 (SD 9.0) points out of 73. Mean energy intake was 8609 (SD 2382) kJ/day with nutrient‐dense core foods contributing 65.9% and energy‐dense, nutrient‐poor, non‐core foods contributing 34.1% of total energy intake.

### 3.2 Associations Between Sleep Quality and Dietary Variables

Associations between sleep quality (PSQI global score) and dietary variables are shown in Table 2 and Supporting Information S1: Table 1.

**Table 2:** Adjusted analysis for the association between sleep quality and dietary variables (*n* = 1070).

Dietary variable	Coefficient (SE)	95% CI	*p*‐value
ARFS^†^	−0.203 (0.106)	−0.412, 0.005	0.056
Energy (kJ/day)^‡^	1.004 (0.003)	0.998, 1.011	0.197
Core food (%EI)^†^	−0.221 (0.127)	−0.470, 0.029	0.083
Non‐core food (%EI)^†^	0.220 (0.127)	−0.029, 0.470	0.083
Protein (%EI)^‡^	1.001 (0.002)	0.998, 1.005	0.417
Carbohydrates (%EI)^†^	−0.012 (0.069)	−0.148, 0.124	0.859
Total fat (%EI)^†^	0.042 (0.052)	−0.061, 0.144	0.428
Saturated fat (%EI)^§^	1.004 (0.002)	1.000, 1.008	**0.046**
Fibre (g/day)^‡^	0.998 (0.004)	0.990, 1.005	0.578
Iron (mg/day)^‡^	1.000 (0.004)	0.993, 1.007	0.992
Iodine (µg/day)^‡^	1.007 (0.005)	0.998, 1.017	0.125
Zinc (mg/day)^‡^	1.004 (0.004)	0.997, 1.011	0.303
Folate eq. (µg/day)^‡^	1.003 (0.004)	0.995, 1.011	0.500

*Notes:* The model family and link used were noted for each outcome. All models were adjusted for relevant confounders including age, household income, pre‐pregnancy BMI, physical activity, DASS, cohort. Bold value are statistically significant.

† Generalised Linear Model with Gaussian family and Identity link.

‡ Generalised Linear Model with Gamma family and Log link.

§ Generalised Linear Model with Gaussian family and Log link.

In unadjusted analyses, poorer sleep quality (higher PSQI score) was associated with lower overall diet quality (ARFS score) (Exp(β) = −0.292, 95% CI: −0.488, −0.096, *p* = 0.004), higher total energy intake (Exp(β) = 1.006, 95% CI: 1.000, 1.012, *p* = 0.049), lower percentage of total energy from nutrient‐dense, core foods (Exp(β) = −0.365, 95% CI: −0.600, −0.131, *p* = 0.002), higher percentage of energy from non‐core foods (Exp(β) = 0.365, 95% CI: 0.130, 0.600, *p* = 0.002), and higher percentage of energy from saturated fat (Exp(β) = 1.004, 95% CI: 1.000, 1.008, *p* = 0.031).

In the adjusted analyses, only the relationship with saturated fat intake remained statistically significant. Specifically, each one‐point increase in PSQI global score (indicating poorer sleep quality), was associated with a 0.4% increase in saturated fat intake as a proportion of total energy (Exp(β) = 1.004, 95% CI: 1.000, 1.008, *p* = 0.046). Overall, adjustment for confounders attenuated the associations between sleep quality and dietary variables. No significant associations were observed between sleep quality and other nutrients (i.e., percentage of energy intake from protein, carbohydrates and total fat, fibre, iron, iodine, zinc, or folate equivalents) in either the unadjusted or adjusted models.

### 3.3 Associations Between Sleep Duration and Dietary Variables

Associations between sleep duration and dietary variables are presented in Table 3 and Supporting Table 2. Normal sleep duration (i.e., 7–9 h/day for adults aged 18–64 years) served as the reference category (Hirshkowitz et al. 2015).

**Table 3:** Adjusted analysis for the association between sleep duration and dietary variables (*n* = 1070).

Dietary variable	Short sleep vs. normal sleep			Long sleep vs. normal sleep		
	Coefficient (SE)	95% CI	*p*‐value	Coefficient (SE)	95% CI	*p*‐value
ARFS^†^	−2.600 (0.686)	−3.947, −1.254	**< 0.001**	−0.036 (1.578)	−3.132, 3.060	0.982
Energy (kJ/day)^‡^	0.971 (0.021)	0.931, 1.012	0.163	1.048 (0.049)	0.954, 1.154	0.339
Core food (%EI)^†^	−1.534 (0.824)	−3.152, 0.084	0.063	−2.608 (1.896)	−6.328, 1.113	0.169
Non‐core food (%EI)^†^	1.533 (0.824)	−0.084, 3.151	0.063	2.606 (1.896)	−1.114, 6.325	0.170
Protein (%EI)^‡^	1.006 (0.012)	0.983, 1.030	0.624	1.014 (0.028)	0.961, 1.071	0.610
Carbohydrates (%EI)^†^	−0.259 (0.450)	−1.142, 0.624	0.565	0.515 (1.035)	−2.545, 1.516	0.619
Total fat (%EI)^†^	0.261 (0.340)	−0.405, 0.928	0.442	0.490 (0.781)	−1.042, 2.023	0.530
Saturated fat (%EI)^†^	0.102 (0.181)	−0.253, 0.458	0.572	0.461 (0.417)	−0.356, 1.279	0.268
Fibre (g/day)^‡^	0.945 (0.025)	0.900, 0.992	**0.022**	1.004 (0.057)	0.900, 1.124	0.941
Iron (mg/day)^‡^	0.962 (0.023)	0.919, 1.006	0.090	1.046 (0.053)	0.944, 1.163	0.394
Iodine (µg/day)^‡^	0.967 (0.031)	0.911, 1.027	0.272	1.113 (0.070)	0.972, 1.282	0.127
Zinc (mg/day)^‡^	0.967 (0.024)	0.924, 1.013	0.158	1.057 (0.054)	0.952, 1.178	0.304
Folate eq. (µg/day)^‡^	0.968 (0.026)	0.921, 1.019	0.213	1.083 (0.059)	0.966, 1.220	0.178

*Note:* The model family and link used were noted for each outcome. All models were adjusted for relevant confounders including age, household income, pre‐pregnancy BMI, physical activity, DASS, cohort. Normal sleep was defined as 7–9 h per day, short sleep duration was defined as < 7 h per day, long sleep was defined as > 9 h per day. bold value are statistically significant.

† Generalised Linear Model with Gaussian family and Identity link.

‡ Generalised Linear Model with Gamma family and Log link.

In unadjusted models, compared to women with normal sleep duration, short sleep (< 7 h/day) was associated with lower diet quality (β = −2.773, 95% CI: −4.093, −1.453, *p* < 0.001) and lower fibre intake (Exp(β) = 0.950, 95% CI: 0.906, 0.997, *p* = 0.037). The unadjusted analyses also found that both short and long sleep duration were associated with lower core food intake (Short: [Exp(β) = −1.786, 95% CI: −3.374, −0.197, *p* = 0.028]; Long: [Exp(β) = −3.826, 95% CI: −7.576, −0.075, *p* = 0.046]), and higher non‐core food intake (Short: [Exp(β) = 1.784, 95% CI: 0.196, 3.373, *p* = 0.028]; Long: [Exp(β) = 3.824, 95% CI: 0.074, 7.575, *p* = 0.046]).

After adjustments for confounders, short sleep duration remained significantly associated with lower diet quality (*β* = −2.600, 95% CI: −3.947, −1.254, *p* < 0.001) and lower fibre intake (Exp(β) = 0.945, 95% CI: 0.900, 0.992, *p* = 0.022) compared to normal sleep. This corresponds to an average 2.6‐point lower diet quality score and approximately 5.5% lower fibre intake for short sleepers. The associations between short and long sleep duration and core/non‐core food intake were attenuated and no longer significant (*p* > 0.05) after adjustment. No other significant associations were observed between sleep duration and other dietary variables (i.e., total energy, percentage of energy from protein, carbohydrates and total fat, saturated fat, iron, iodine, zinc, or folate equivalents) in either the unadjusted or adjusted models (*p* > 0.05).

## 4 Discussion

This study examined associations between sleep quality and duration and dietary variables in Australian pregnant women. After adjusting for confounders, poorer sleep quality was associated with higher saturated fat intake as a proportion of total energy intake, while short sleep duration (< 7 h per night) was associated with lower overall diet quality and lower fibre intake.

The finding that poorer sleep quality was associated with higher saturated fat intake in this sample of pregnant women provides new insights into the relationship between sleep quality and dietary intake. Evidence in pregnancy remains limited; a 2023 systematic review of 25 studies that examined associations between sleep and diet in pregnant women identified only two examining fat intake, neither of which specifically assessed saturated fat, and findings were inconsistent (von Ash et al. 2023). For example, Chang et al. reported higher total fat consumption with sleep disturbances in pregnant women (Chang et al. 2015), whereas Bennett et al. observed lower monosaturated fat intake in those with poorer sleep (Bennett et al. 2019). Although the result from the current study is statistically significant, the magnitude of the effect was small, with a 0.4% increase in energy from saturated fat for each one‐point increase in PSQI score. However, its clinical relevance should be considered in context. In this cohort, mean saturated fat intake (14% of total energy) exceeded current recommendations (≤ 10% total energy), suggesting that even small increases associated with poorer sleep may further widen this gap. While the effect size is small at an individual level, it may therefore be more meaningful at a population level.

Pregnant women are advised by national guidelines to limit saturated fat intake because of its links to complications such as preeclampsia, gestational diabetes mellitus and preterm delivery (Khaire et al. 2020). Excessive intake of saturated fat can impair maternal lipid metabolism and glucose tolerance, which may negatively impact placental function, foetal growth and increase susceptibility of metabolic diseases in offspring (Khaire et al. 2020). However, the women in this sample reported mean saturated fat intake (14% EI) that exceeds dietary recommendations (≤ 10% EI), thereby increasing the risk for adverse pregnancy and birth outcomes. While the data did not specify the food sources, the excessive saturated fat intake was likely derived from non‐core foods. This is supported by the unadjusted association between poor sleep quality and higher non‐core food intake, though this association was attenuated after adjustment for confounders. Given the observed association between poorer sleep quality and higher saturated fat intake, sleep quality may be an important factor to consider when examining adherence to dietary guidelines and maternal and infant health outcomes. Conversely, improving diet quality may also enhance sleep, which is associated with reduced risk of cardiometabolic complications such as hypertension and preeclampsia. It is also important to consider that the relationship between sleep and diet is likely bidirectional, with emerging evidence suggesting that dietary patterns and nutrient intake may also influence sleep quality and duration (Bazzani and Faraguna 2025). These bidirectional pathways warrant further investigation to inform integrated interventions targeting both sleep and diet during pregnancy.

A significant relationship was observed between sleep quality and overall diet quality in the unadjusted analysis but became non‐significant after adjustment (*p* = 0.056). There is very limited research on this relationship, but the absence of a statistically significant association between sleep quality and diet quality in the current study contrasts with the findings of Van Lee et al. in the GUSTO cohort, where a positive correlation between sleep quality and overall diet quality was reported (van Lee et al. 2017). However, the significant association they observed was attenuated and became non‐significant (*p* = 0.096) after further adjustment for anxiety scores (van Lee et al. 2017), which were also adjusted for in the current study. To extend the literature, further research is needed to clarify the associations between sleep quality and diet quality, as it is critical for supporting the health and wellbeing of both pregnant women and their infants.

The current analysis also observed independent associations between short sleep duration and lower diet quality and fibre intake, suggesting that sleep duration may influence dietary behaviours in pregnant women. Short sleepers scored, on average, 2.6 points lower ARFS compared to those with normal sleep duration, reflecting lower variety in nutrient‐dense core foods such as whole grains, fruits and vegetables. Although these associations were statistically significant, the magnitude of the effects was modest. However, their clinical relevance should be considered in context. A 2.6‐point reduction in ARFS represents a meaningful shift within diet quality categories, particularly in a cohort with an overall mean score indicative of suboptimal diet quality. Similarly, the observed reduction in fibre intake (~5.5%) may be important, given the role of fibre in supporting maternal gastrointestinal function, metabolic health, and fetal development, especially where baseline intake may already be inadequate (Pretorius and Palmer 2021; Reynolds et al. 2019).

Several physiological and behavioural mechanisms may explain these relationships (Dashti et al. 2015). In general populations, sleep restriction alters hunger‐regulating hormones, elevating ghrelin and suppressing leptin, which may promote emotional eating and greater intake of high‐fat, high‐sugar foods (Agrisdian et al. 2022; Dakanalis et al. 2023; Fariandini et al. 2022). Also, sleep restriction stimulates the hedonic mechanism controlled by brain reward pathways, affecting food decisions and increased preference for energy‐dense snacks (Dashti et al. 2015). From a behavioural perspective, shorter sleep duration creates additional eating windows due to increased awakening times, often in the form of convenient carbohydrate sources and energy‐dense snacks, displacing nutrient‐dense healthy core foods (Dashti et al. 2015).

In addition to these pathways, emerging evidence suggests that sleep and dietary patterns may also interact through alterations in the gut microbiome, providing an additional biological pathway (Sejbuk et al. 2024). Both poor sleep and suboptimal dietary patterns have been associated with reduced microbial diversity and shifts in microbial composition, which may in turn influence metabolic processes, including those that influence systemic inflammation (Randeni et al. 2024; Sun et al. 2023). This highlights the potential for future studies evaluating the role of the microbiome as a mediator linking sleep, diet, and maternal health outcomes.

Furthermore, given that poor diet quality during pregnancy is associated with higher gestational weight gain, gestational hypertension (Abdollahi et al. 2021), lower birth weight (Chia et al. 2019), and preterm birth (Chia et al. 2019), the observed association between short sleep duration and poorer dietary outcomes highlights sleep duration as a potentially important correlate of dietary behaviours during pregnancy that warrants further investigation. These findings highlight the importance of considering sleep quality and sleep duration within antenatal care, given their observed associations with dietary outcomes.

Contrary to findings from meta‐analyses in general adult populations (Al Khatib et al. 2017; Fenton et al. 2021), the current study found no association between short sleep duration and higher energy intake. However, direct comparison is limited because those meta‐analyses were conducted in studies with sleep duration of ≤ 5.5 h per day (Al Khatib et al. 2017; Fenton et al. 2021), whereas the current study used a threshold of < 7 h per day, consistent with national sleep recommendations (Hirshkowitz et al. 2015). Findings from this study indicate that short sleep duration was associated with differences in the nutritional quality of food consumed by pregnant women, rather than the overall quantity of food. Specifically, poorer sleep coincided with lower diet quality and fibre intake, without an increase in total energy intake. This interpretation aligns with evidence from both pregnant and non‐pregnant populations, which link poor sleep quality, short sleep duration, and increased nighttime disturbances with poorer prenatal eating behaviours (Pauley et al. 2023).

This study provides novel insights into the relationship between sleep quality, sleep duration, and dietary patterns during pregnancy. The high prevalence of poor sleep quality observed in this and previous studies (Christian et al. 2019; Mindell et al. 2015), may partially explain the widespread challenges in dietary guidelines adherence among pregnant women (Caut et al. 2020). These findings highlight the potential value of considering sleep quality and sleep duration alongside dietary behaviours in antenatal care and future research. However, longitudinal studies are required to establish causality, and intervention trials, where possible, should evaluate whether sleep‐diet strategies can improve dietary patterns and reduce complications such as excessive gestational weight gain and preeclampsia, which have long‐term health consequences (Neiger 2017).

A key strength of this study is the large sample size drawn from two Australian pregnancy cohorts, which supports the generalisability of the findings to similar Western, higher‐income populations. The comprehensive analysis of diet quality and dietary intake extends existing literature on the relationship between sleep and maternal nutrition. The use of validated tools, the PSQI for sleep quality and duration, AES and ARFS for dietary variables, improves reliability and comparability of the data. Further, the timing of data collection at 18–20 weeks gestation is beyond the first trimester, when hyperemesis and atypical food choices are most common, making the findings more representative of usual dietary patterns during the remainder of pregnancy. However, this timing does not capture changes that occur later in pregnancy, when sleep may deteriorate further (Sedov et al. 2018). Adjustment for key confounders, including socioeconomic status and mental health indicators, further strengthens the validity of the observed associations.

However, several limitations should be considered. The cross‐sectional design precludes causal inference. As this study is observational and cross‐sectional in design, cause‐and‐effect relationships cannot be established. Sleep and dietary behaviours are likely to be bidirectionally related; although the current study examined sleep as the primary exposure, it is plausible that dietary intake may also influence sleep quality and duration. Emerging evidence suggests that dietary patterns, macronutrient composition, and meal timing may affect sleep outcomes (Bazzani and Faraguna 2025). Future longitudinal studies are needed to clarify these temporal and causal relationships, as intervention studies may be ethically challenging in pregnant women. Residual confounding may also remain due to unmeasured factors such as chronotype, social jetlag and shift work, which may influence both sleep and dietary behaviours (Cheng and Chan 2025). Sleep and dietary data were self‐reported, introducing potential recall and social desirability bias (Tang et al. 2022). In addition, sleep was assessed using the PSQI, which, although widely used, is a self‐report instrument and hence may not fully capture pregnancy‐specific sleep disturbances such as nocturia, restless legs syndrome, and sleep‐disordered breathing. The use of objective measures (e.g., actigraphy) or pregnancy‐specific sleep assessment tools may provide more precise estimates of sleep patterns and could yield different associations with dietary outcomes. Finally, generalisability beyond similar Western, higher‐income contexts may be limited, as most participants were from relatively socioeconomically advantaged backgrounds and were born in Australia, the United Kingdom, or New Zealand. Aboriginal and Torres Strait Islander women and individuals from lower socioeconomic backgrounds were underrepresented in the current study sample, and findings should therefore be interpreted with caution when extrapolating to more culturally and socioeconomically diverse populations, where sleep patterns, dietary behaviours, and related constraints may differ substantially. Findings may not apply to populations with different cultural, dietary, and sleep practices, or to more ethnically diverse or lower income groups.

## 5 Conclusion

This study contributes to the limited evidence on the relationship between sleep quality, sleep duration, and diet during pregnancy. In this sample of women, poorer sleep quality was associated with higher saturated fat intake, while short sleep duration was linked to lower overall diet quality and reduced fibre intake. No significant associations were observed with total energy intake, which suggests that in pregnant women, sleep may influence the quality rather than the quantity of dietary intake. These findings underscore the importance of considering sleep quality and sleep duration as factors associated with maternal nutrition during pregnancy. Future research should prioritise longitudinal studies to establish temporality and causality between sleep quality, sleep duration, and dietary outcomes during pregnancy.

## Author Contributions

S.F. and M.J.D. contributed to study conceptualisation. S.F. and M.J.D. developed the analysis plan. G.L.H.C. performed the formal analysis. G.L.H.C. and S.F. prepared the original draft. S.F., M.J.D and C.E.C. provided supervision. All authors contributed to the interpretation of study results and critical revision and editing of the manuscript, and all have approved the final version of the manuscript. This study was undertaken as a partial requirement for the award of the degree of Bachelor of Nutrition and Dietetics (Honours programme) at the University of Newcastle, Australia.

## Conflicts of Interest

The authors declare no conflicts of interest.

## Supporting information


Supporting File

## Data Availability

The data used in this study is not publicly available due to the rules and restrictions of the approving Ethics Committees.
