# Supplementary material for: Associations Between Sleep Health and Diet Quality and Dietary Intake During Pregnancy: Evidence From Two Australian Cohorts
Source: Matern Child Nutr. 2026 Sep 21;22(3):e70256. doi: 10.1111/mcn.70256 (PMC13591229; doi:10.1111/mcn.70256)
Supplement: Supplementary file 1 [file MCN-22-e70256-s001.docx]

**Associations between sleep health and diet quality and dietary intake during pregnancy: Evidence from two Australian cohorts.**

**Supplementary material**

**Supplementary table 1: Unadjusted analysis for the association between sleep quality (PSQI global score) and dietary variables (n = 1,070)**

| **Dietary variable** | **Coefficient (SE)** | **95% CI** | **p-value** |
| --- | --- | --- | --- |
| ARFS ^†^ | -0.292 (0.100) | -0.488, -0.096 | **0.004** |
| Energy (kJ/day) ^‡^ | 1.006 (0.003) | 1.000, 1.012 | **0.049** |
| Core Food (%EI) ^†^ | -0.365 (0.120) | -0.600, -0.131 | **0.002** |
| Non-Core food (%EI) ^†^ | 0.365 (0.120) | 0.130, 0.600 | **0.002** |
| Protein (%EI) ^‡^ | 1.001 (0.002) | 0.998, 1.004 | 0.564 |
| Carbohydrates (%EI) ^†^ | -0.004 (0.065) | -0.130, 0.123 | 0.957 |
| Total Fat (%EI) ^†^ | 0.048 (0.048) | -0.047, 0.143 | 0.326 |
| Saturated Fat (%EI) ^§^ | 1.004 (0.002) | 1.000, 1.008 | **0.031** |
| Fibre (g/day) ^‡^ | 0.998 (0.004) | 0.991, 1.005 | 0.630 |
| Iron (mg/day) ^‡^ | 1.001 (0.003) | 0.994, 1.008 | 0.792 |
| Iodine (µg/day) ^‡^ | 1.007 (0.005) | 0.998, 1.016 | 0.126 |
| Zinc (mg/day) ^‡^ | 1.005 (0.003) | 0.998, 1.012 | 0.135 |
| Folate eq. (µg/day) ^‡^ | 1.003 (0.004) | 0.996, 1.011 | 0.389 |

Notes: The model family and link used were noted for each outcome. ^†^ Generalised Linear Model with Gaussian family and Identity link; ^‡^ Generalised Linear Model with Gamma family and Log link; ^§^ Generalised Linear Model with Gaussian family and Log link.

**Supplementary Table 2: Unadjusted analysis for the association between sleep duration and dietary variables (n = 1,070)**

| **Dietary variable** | **Short Sleep vs Normal Sleep** | | | **Long Sleep vs Normal Sleep** | | |
| --- | --- | --- | --- | --- | --- | --- |
|  | **Coefficient (SE)** | **95% CI** | **p-value** | **Coefficient (SE)** | **95% CI** | **p-value** |
| ARFS ^†^ | -2.773 (0.673) | -4.093,  -1.453 | **<0.001** | -1.045 (1.588) | -4.161, 2.070 | 0.510 |
| Energy (kJ/day) ^‡^ | 0.992 (0.021) | 0.952, 1.033 | 0.692 | 1.044 (0.049) | 0.949, 1.151 | 0.382 |
| Core food (%EI) ^†^ | -1.786 (0.810) | -3.374,  -0.197 | **0.028** | -3.826 (1.911) | -7.576,  -0.075 | **0.046** |
| Non-core food (%EI) ^†^ | 1.784 (0.810) | 0.196, 3.373 | **0.028** | 3.824 (1.911) | 0.074, 7.575 | **0.046** |
| Protein (%EI) ^‡^ | 1.008 (0.012) | 0.985, 1.031 | 0.508 | 1.011 (0.028) | 0.959, 1.068 | 0.686 |
| Carbohydrates (%EI) ^†^ | -0.348 (0.436) | -1.203, 0.508 | 0.425 | -0.205 (1.029) | -2.224, 1.814 | 0.842 |
| Total Fat (%EI) ^†^ | 0.388 (0.327) | -0.254, 1.030 | 0.236 | 0.379 (0.773) | -1.138, 1.895 | 0.624 |
| Saturated Fat (%EI) ^†^ | 0.199 (0.176) | -0.146, 0.543 | 0.259 | 0.472 (0.415) | -0.342. 1.286 | 0.255 |
| Fibre (g/day) ^‡^ | 0.950 (0.024) | 0.906, 0.997 | **0.037** | 0.980 (0.058) | 0.877, 1.099 | 0.720 |
| Iron (mg/day) ^‡^ | 0.973 (0.023) | 0.930, 1.017 | 0.222 | 1.030 (0.054) | 0.929, 1.146 | 0.582 |
| Iodine (µg/day) ^‡^ | 0.991 (0.030) | 0.934, 1.052 | 0.767 | 1.093 (0.072) | 0.953, 1.262 | 0.214 |
| Zinc (mg/day) ^‡^ | 0.989 (0.023) | 0.945, 1.035 | 0.629 | 1.050 (0.055) | 0.945, 1.171 | 0.374 |
| Folate eq. (µg/day) ^‡^ | 0.980 (0.025) | 0.933, 1.030 | 0.428 | 1.067 (0.060) | 0.951, 1.203 | 0.276 |

Notes: The model family and link used were noted for each outcome. ^†^ Generalised Linear Model with Gaussian family and Identity link; ^‡^ Generalised Linear Model with Gamma family and Log link. Normal sleep was defined as 7-9 hours per day, short sleep duration was defined as <7 hours per day, long sleep was defined as >9 hours per day.
